# Brain Stimulation Techniques in Research and Clinical Practice: A Comprehensive Review of Applications and Therapeutic Potential in Parkinson’s Disease

**DOI:** 10.3390/brainsci15010020

**Published:** 2024-12-27

**Authors:** Ata Jahangir Moshayedi, Tahmineh Mokhtari, Mehran Emadi Andani

**Affiliations:** 1 School of Information Engineering, Jiangxi University of Science and Technology, Ganzhou 341000, China; ajm@jxust.edu.cn; 2 Department of Entomology and Nematology, UCD Comprehensive Cancer Center, University of California, Davis, CA 95616, USA; tmokhtari@ucdavis.edu; 3 Department of Neurosciences, Biomedicine and Movement Sciences, University of Verona, Via Casorati, 37131 Verona, Italy

**Keywords:** Parkinson’s disease, brain stimulation, transcranial magnetic stimulation, transcranial electrical stimulation, transcranial focused ultrasound stimulation, transcutaneous vagus nerve stimulation, deep brain stimulation

## Abstract

Parkinson’s Disease (PD) is a progressive neurodegenerative disorder characterized by a range of motor and non-motor symptoms (NMSs) that significantly impact patients’ quality of life. This review aims to synthesize the current literature on the application of brain stimulation techniques, including non-invasive methods such as transcranial magnetic stimulation (TMS), transcranial electrical stimulation (tES), transcranial focused ultrasound stimulation (tFUS), and transcutaneous vagus nerve stimulation (tVNS), as well as invasive approaches like deep brain stimulation (DBS). We explore the efficacy and safety profiles of these techniques in alleviating both motor impairments, such as bradykinesia and rigidity, and non-motor symptoms, including cognitive decline, depression, and impulse control disorders. Current findings indicate that while non-invasive techniques present a favorable safety profile and are effective for milder symptoms, invasive methods like DBS provide significant relief for severe cases that are unresponsive to other treatments. Future research is needed to optimize stimulation parameters, establish robust clinical protocols, and expand the application of these technologies across various stages of PD. This review underscores the potential of brain stimulation as a vital therapeutic tool in managing PD, paving the way for enhanced treatment strategies and improved patient outcomes.

## 1. Introduction

Parkinson’s Disease (PD) is a progressive neurological disorder that significantly impacts quality of life (QOL), affecting movement and causing symptoms such as tremors, rigidity, and bradykinesia (slowness of movement). As the disease progresses, it can lead to severe disability, affecting daily activities and requiring long-term care [1]. As life expectancy rises, the number of individuals with PD is expected to grow, highlighting the need for effective treatments and support [2]. PD is staged using the Hoehn and Yahr scale, which includes five stages [3]. Stage 1 involves unilateral symptoms like tremors, stiffness, or slowness on one side of the body. Stage 2 features bilateral symptoms, with worsening motor difficulties, but the person remains independent. Stage 3 shows balance impairment and mild to moderate disability, with some assistance needed for daily activities. Stage 4 involves severe disability, including difficulty walking or standing unassisted, requiring help with daily tasks. Stage 5 is the most advanced, with severe impairment leading to wheelchair or bed use. These stages help assess disease progression and plan appropriate treatment [4]. α-Synuclein is a key protein involved in PD’s progressive neurodegeneration, spreading from the peripheral nervous system to the brain [5].

Brain stimulation methods have been an area of significant interest and development in both research and clinical practice for several decades [6]. These techniques, which involve the application of electrical or magnetic stimuli to the brain, aim to modulate neural activity in targeted regions [7]. The development and refinement of these methods have opened new avenues for understanding brain function and treating various neurological and psychiatric disorders [8]. For over three decades, deep brain stimulation (DBS) has remained a viable treatment option for PD. Nevertheless, this treatment is not fully utilized, primarily because of widespread misinformation concerning its associated risks and clinical results [1]. Furthermore, the most effective stimulation approaches for freezing of gait (FOG), non-motor symptoms (NMSs), and the best timing for DBS are still being studied [2]. The application of brain stimulation techniques, encompassing both non-invasive and invasive modalities, represents a promising frontier in the management of PD. On the other hand, various invasive and non-invasive methods of brain stimulation have been introduced to treat patients with PD across varying levels of severity [4].

The organization of this paper is as follows: We begin with an overview of brain stimulation techniques, distinguishing between non-invasive brain stimulation (NIBS), which includes Transcranial Magnetic Stimulation (TMS), Transcranial Electrical Stimulation (tES), Transcranial Focused Ultrasound Stimulation (tFUS), and Transcutaneous Vagal Nerve Stimulation (tVNS), and invasive brain stimulation (IBS), particularly DBS. We then explore their clinical applications in managing both motor and NMSs of PD. The discussion summarizes our findings, addresses clinical implications, and identifies challenges in current research. Finally, we outline gaps in the literature and suggest avenues for future investigation to enhance the role of brain stimulation in PD management.

In this comprehensive review, we aim to investigate and compare various brain stimulation methods for individuals with PD, highlighting their respective advantages and disadvantages. The primary objectives of this paper are as follows:

- Review Brain Stimulation Techniques: Provide an overview of non-invasive (TMS, tES, tFUS, tVNS) and invasive (DBS) brain stimulation methods and their therapeutic applications.
- Investigate the Effectiveness of Brain Stimulation on Motor Symptoms of PD.
- Evaluate the Effectiveness of Brain Stimulation on Non-Motor Symptoms of PD.
- Compare Safety Profiles.
- Identify Future Research Directions.

## 2. Materials and Methods

**Figure 1:**
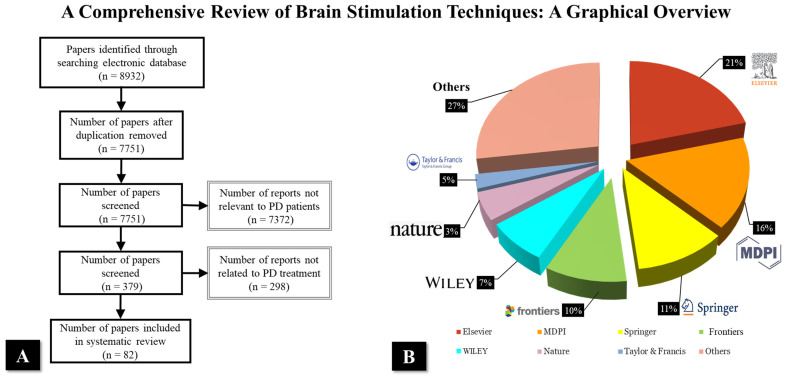
The reviewed paper analyses: (**A**) review Prisma diagram, and (**B**) the papers based on publishers.

This comprehensive review was conducted to provide a broad synthesis of the current literature on brain stimulation techniques in PD. The methodology was designed to ensure a thorough exploration of the topic while maintaining flexibility to include diverse studies relevant to the therapeutic applications of brain stimulation. To gather relevant literature, a structured search was performed across several reputable databases and publisher websites, including PubMed, ScienceDirect, Springer, MDPI, Frontiers, and Wiley Online Library. The search was conducted using a combination of carefully selected keywords, such as “Parkinson’s disease”, “Non-invasive brain stimulation”, “Therapeutic potential”, “Brain stimulation techniques”, and “Parkinson’s treatment”. The search was restricted to peer-reviewed articles published in English, with a focus on studies published in the last ten years (i.e., between 2015 and 2024). However, particular emphasis was placed on more recent publications (2020–2024) to ensure the inclusion of the latest advancements in the field. The selection of articles was guided by specific inclusion and exclusion criteria. Reviews, meta-analyses, and original research articles published in peer-reviewed journals were prioritized. Conversely, studies not directly related to Parkinson’s disease or brain stimulation techniques, articles focusing solely on animal models without translational relevance, and non-peer-reviewed materials such as conference abstracts or editorials were excluded. The reviewed paper analyses are shown in Figure 1, which shows the publication distribution of the 82 reviewed papers, categorized by publisher and year of publication. In terms of publishers, the majority of the papers were published by Elsevier, accounting for 20.99%, followed by MDPI with 16.05%, and Springer with 11.11%. Other notable publishers include Frontiers (9.88%), Wiley (7.41%), and Nature (4.94%). A smaller portion of papers, 2.47%, were published by Taylor & Francis, while 27.16% were classified under ’Others’. Regarding the year of publication, the majority of the papers (91.36%) were published since 2020, with a total of 74 papers, while seven papers (20.99%) were published before 2020.

The authors believe that this review can serve as a valuable resource for researchers by providing a comprehensive understanding of the various brain stimulation techniques and their applications in both research and clinical practice. It can help researchers identify the therapeutic potential of these methods, guide the design of future studies, and inform the development of new treatments for neurological and psychiatric disorders. Additionally, the review offers insights into safety considerations, current trends, and innovations in the field, enabling researchers to stay up-to-date with the latest advancements. Ultimately, the authors believe that this review will inspire further research, promote interdisciplinary collaboration, and help advance the clinical use of brain stimulation techniques.

## 3. Results

### 3.1. Development of Various Brain Stimulation Methods

Brain stimulation methods can be divided into IBS and NIBS. NIBS methods are particularly proposed for PD due to their safety, as they avoid surgery or skin penetration. They generally have minimal side effects compared to invasive treatments [9]. These techniques allow targeted stimulation of brain regions involved in motor control, improving symptom management [10]. They are accessible in outpatient settings and can be adjusted to fit individual patient needs. Additionally, they can complement other treatments, enhancing overall symptom control and quality of life [10].

**Figure 2:**
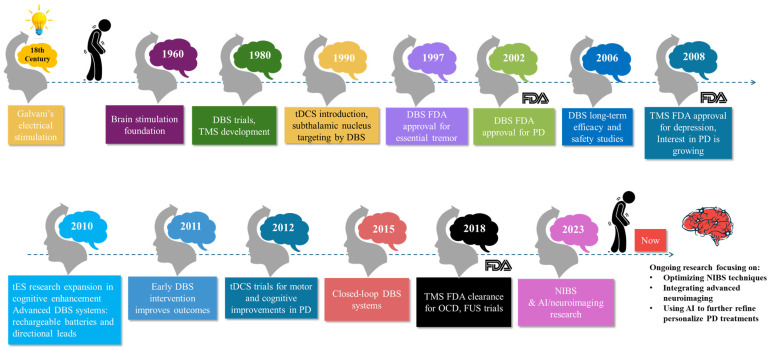
Timeline of brain stimulation methods for the treatment of PD.

In the 11th century, Ibn Sidah, a renowned physician, proposed the use of a live electric catfish as a treatment for epilepsy [11]. The timeline of brain stimulation in PD (Figure 2) begins in the 18th century with Luigi Galvani’s pioneering work on the electrical stimulation of animal tissues, which laid the groundwork for the field of electrophysiology and the concept of bioelectricity [12]. In the 1960s, initial observations suggested that lesions in the basal ganglia could alleviate symptoms of PD, paving the way for future brain stimulation techniques [12]. The 1980s marked the commencement of early trials exploring the application of DBS. This decade also witnessed the development of TMS, providing a non-invasive method to stimulate the human brain and allowing researchers to study brain–behavior relationships. The 1990s saw the introduction of transcranial direct current stimulation (tDCS), offering a simple, portable, and non-invasive method to modulate cortical excitability. Significant advancements in DBS techniques were observed during this time, particularly with the targeting of the subthalamic nucleus in 1993, which demonstrated substantial improvements in the motor symptoms of patients with PD, further solidifying the role of neurostimulation in managing neurological conditions.

In 1997, DBS received its first FDA approval for the treatment of essential tremor, marking its acceptance as a viable therapeutic option [10]. By 2002, it gained FDA approval for PD, solidifying its role in managing advanced motor symptoms. By 2006, long-term studies began to demonstrate the efficacy and safety of DBS in managing motor symptoms and improving quality of life in PD patients. In 2008, TMS was approved by the FDA for the treatment of major depressive disorder, highlighting its potential as a non-invasive treatment for psychiatric conditions. Pilot studies demonstrated that repetitive stimulation could improve motor function and reduce tremors in PD patients, leading to a growing interest in non-invasive methods [13]. During the 2010s, the use of tES expanded in research, focusing on its potential to enhance cognitive functions and aid in neurorehabilitation. In 2010, more advanced DBS systems with rechargeable batteries and directional leads were introduced, allowing for more precise targeting and customization of stimulation [14]. Studies conducted in 2011 demonstrated that early intervention with DBS in PD patients could lead to better outcomes compared to those who received stimulation later in the disease course. In 2012, clinical trials investigating the effects of tDCS on motor symptoms and cognitive function in PD patients yielded promising results. By 2015, clinical trials began exploring closed-loop DBS systems, which could adjust stimulation in real time based on neural feedback, potentially improving efficacy and reducing side effects. The combination of TMC and tDCS with other therapies, such as physical therapy and medication, was also studied to maximize therapeutic outcomes, providing synergistic benefits and potentially reducing the need for higher medication dosages. In 2018, TMC received FDA clearance for treating obsessive-compulsive disorder, further broadening its clinical applications [15]. Initial clinical trials explored the use of focused ultrasound stimulation for targeting specific brain regions without surgical intervention, showing potential in alleviating tremors and improving motor function in PD patients.

Entering the 2020s, advancements in adaptive and personalized NIBS approaches gained momentum. Adaptive TMS systems, capable of dynamically adjusting stimulation parameters based on real-time neural feedback, were developed, offering more tailored and effective treatments for PD patients. Researchers also began exploring the combination of NIBS with emerging therapies such as gene therapy and stem cell therapy, aiming to enhance treatment outcomes and provide long-term benefits for individuals with PD. By 2023, ongoing research into NIBS techniques, including TMS and tFUS, sought to provide alternative or complementary treatments to traditional DBS. Studies investigated the potential of combining TMS, tDCS, and FUS with advanced neuroimaging and artificial intelligence to further refine and personalize treatments. TMS is being researched for its potential benefits in PD, particularly for NMSs such as depression, while tDCS is under investigation for its effects on enhancing motor function and cognitive abilities in PD patients [16]. Currently, DBS serves as a primary treatment for advanced PD, effectively managing motor symptoms. Ongoing advancements focus on optimizing these technologies, including the development of more sophisticated closed-loop DBS systems and the integration of artificial intelligence to improve the precision and effectiveness of brain stimulation for PD. Looking towards the future, there is an increased understanding of the underlying mechanisms of DBS and its long-term effects on the brain and overall health of PD patients. Researchers are actively exploring new brain targets and personalized stimulation protocols tailored to individual patient needs and disease progression, further enhancing the potential of neurostimulation in the treatment of PD.

### 3.2. Brain Stimulation for Treating Parkinson’s Disease

**Figure 3:**
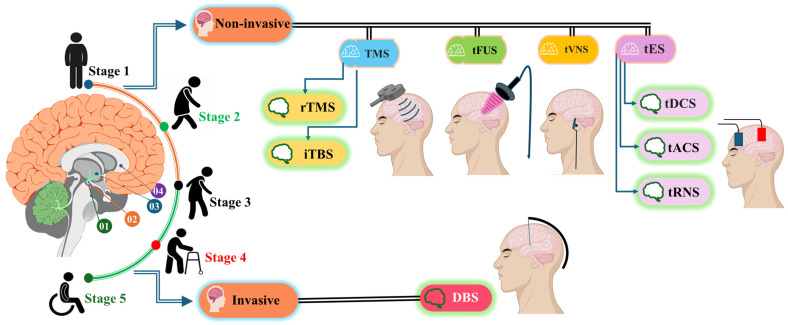
Brain regions relevant to Parkinson’s Disease (PD). The areas highlighted include Substantia Nigra (01), Dopamine Pathway (02), Putamen (Striatum; 03), and Caudate Nucleus (Striatum; 04). The figure illustrates the five stages of PD, along with brain stimulation methods employed for PD patients.

PD symptoms result from the degeneration of dopamine-producing neurons in the substantia nigra, a brain region crucial for motor control (Figure 3). The management of PD involves various medications, including Levodopa/Carbidopa [17], dopamine agonists, MAO-B inhibitors, COMT inhibitors, anticholinergics, and amantadine [18], as well as surgical treatments like DBS and lesioning surgeries [19]. Additionally, physical therapy and exercise can help improve movement, balance, and strength, while lifestyle and supportive therapies including speech therapy, dietary adjustments, and occupational therapy enhance overall well-being [20]. Despite the availability of treatments, there is currently no cure for PD, leading to substantial financial burdens on patients and healthcare systems. Promising advancements in regenerative medicine, including cell engineering, gene therapy, and stem cell therapies, offer hope for future treatments [21]. The introduction of brain stimulation techniques has revolutionized the management of PD, providing significant relief from debilitating motor symptoms and improving patients’ quality of life. Ongoing research and technological advancements are expected to enhance the efficacy and safety of these interventions, bringing hope to those affected by this challenging condition [22]. DBS is particularly vital due to its effectiveness in reducing motor symptoms, improving QOL, and its features of adjustability and reversibility, alongside ongoing advancements in neuromodulation technology [23].

### 3.3. Non-Invasive Technologies

NIBS technologies provide alternative approaches for managing symptoms of PD without the necessity for surgical intervention. These techniques have gained significant interest due to their ability to modulate neural activity with fewer risks compared to invasive methods such as DBS. The primary NIBS technologies include TMS and tDCS [7].

#### 3.3.1. Transcranial Magnetic Stimulation (TMS)

TMS encompasses various techniques, including rTMS and iTBS, all showing promise in treating PD. These NIBS methods target specific brain regions to modulate neural activity and alleviate symptoms [24]. Each technique offers unique advantages, from the general efficacy of TMS to the enhanced and potentially longer-lasting effects of rTMS and iTBS [6].

TMS utilizes magnetic fields generated by a coil placed on the scalp to induce electrical currents in specific brain regions. These magnetic fields penetrate the skull, allowing for modulation of neuronal activity. The frequency and intensity of the magnetic pulses can be adjusted to either excite or inhibit neural circuits. TMS has demonstrated moderate efficacy in improving motor function and reducing symptoms of PD. Its non-invasive nature and general tolerability make it a suitable option for patients who cannot undergo surgery. TMS has shown promise in alleviating both motor symptoms—such as tremors, bradykinesia, and rigidity—and NMSs like depression and cognitive impairments associated with PD. However, the benefits of TMS are often temporary, requiring repeated sessions for sustained effects. Importantly, patient responses to TMS can vary widely, indicating a need for further research to determine optimal stimulation parameters [25].

rTMS involves delivering repeated magnetic pulses at specific frequencies, leading to longer-lasting changes in brain activity compared to single-pulse TMS. In PD, rTMS can be categorized into two main approaches: High-Frequency (HF) rTMS, typically applied at frequencies of 5 Hz or higher, aims to excite neural activity and enhance motor function by stimulating the motor cortex. In contrast, Low-Frequency (LF) rTMS, applied at frequencies of 1 Hz or lower, seeks to inhibit overactive brain regions, potentially reducing tremors and rigidity. rTMS has demonstrated significant benefits in improving motor symptoms and reducing medication needs, while also positively impacting NMSs such as depression and anxiety, thereby enhancing overall quality of life. The non-invasive nature of rTMS makes it a safer option for patients unsuitable for surgery. However, challenges remain, including the need for frequent sessions to maintain benefits and variability in individual patient responses. Additionally, the cost and accessibility of rTMS may hinder widespread adoption [26].

iTBS is a variation of TMS that employs short bursts of high-frequency stimulation patterned to mimic the natural theta rhythm of the brain. iTBS sessions are shorter than traditional rTMS, usually lasting only a few minutes, making it more time-efficient and reducing the treatment burden on patients. Some studies suggest that iTBS can produce similar or even superior outcomes to rTMS, particularly in improving motor function. It is applied to the motor cortex to enhance motor symptoms and has also been explored for cognitive and mood improvements in PD patients. However, limitations exist, as further research is needed to establish the long-term benefits and optimal frequency of iTBS sessions. Responses to iTBS can vary among patients, similar to other forms of TMS [27]. Overall, TBS presents a more time-efficient treatment option with potentially significant outcomes, especially in enhancing motor function, but further investigation is necessary to fully understand its long-term efficacy.

Several studies have investigated the effects of TMS in managing PD [28]. In a review by Nardone et al. (2020) [25], the application of TMS in understanding and treating motor impairments, particularly gait disturbances, was highlighted. The review emphasized the beneficial effects of high-frequency rTMS, especially when applied bilaterally over motor cortical regions, in alleviating motor symptoms in PD. However, it also noted the limited research specifically addressing the effects of rTMS on FOG and other gait disturbances. Furthermore, the review explored the combination of rTMS with treadmill training, suggesting that this could enhance the effectiveness of physical therapy in improving gait. Advanced techniques like H-coil stimulation, which targets deeper brain regions such as the medial prefrontal cortex, were discussed as potential future add-on therapies. Conversely, the review found iTBS to be ineffective in treating gait disturbances in PD [23]. The authors proposed a dual-mode NIBS approach, specifically preconditioning motor cortex rTMS with tDCS, as a novel therapeutic strategy for PD patients experiencing gait disturbances. They also identified the supplementary motor area as a promising target for brain stimulation in FOG treatment. However, they emphasized the need for large-scale, well-designed clinical studies to evaluate the long-term sustainability of these interventions and to optimize stimulation protocols, including target selection, stimulation intensity, duration, and session frequency, to maximize the therapeutic effects of rTMS in PD [24]. Despite their potential benefits, these techniques face challenges such as variability in patient response, temporary effects, and the necessity for frequent sessions [25]. Ongoing research aims to optimize these technologies and improve their efficacy and accessibility, ultimately enhancing outcomes for individuals with PD [29,30].

Zhang et al. (2022) conducted a systematic review and meta-analysis of randomized controlled trials (RCTs) to evaluate the efficacy of rTMS on both motor and NMSs in PD [26]. Their findings indicated that HF rTMS applied to the primary motor cortex (M1) significantly improved motor symptoms and showed potential antidepressant-like effects when targeting the dorsolateral prefrontal cortex (DLPFC). However, the study did not find sufficient evidence supporting cognitive improvement. The authors concluded that rTMS could serve as an effective adjuvant therapy for PD, particularly for enhancing motor symptoms and potentially alleviating depression. They stressed the need for further research to optimize parameters, including stimulation site and frequency, especially concerning its effects on cognitive function and depression in PD patients. Overall, TMS—particularly high-frequency rTMS—shows promise in managing both motor and NMSs in PD, but further studies are required to fully understand its potential in addressing cognitive impairment and depression in this population [26].

#### 3.3.2. Transcranial Electrical Stimulation (tES)

Transcranial electrical stimulation techniques, which include tDCS, tACS, and tRNS, offer promising non-invasive options for managing symptoms of PD. These methods are generally safe and well-tolerated, with the potential to improve motor, cognitive, and mood symptoms. However, they also face challenges such as temporary effects, variability in patient response, and the need for optimized stimulation parameters. Ongoing research aims to enhance the efficacy and accessibility of these techniques, potentially offering new avenues for improving the quality of life for individuals with PD [31].

tDCS involves applying a constant, low-intensity electrical current to the scalp via electrodes. This current can be anodal (positive), known as anodal stimulation, which increases neuronal excitability, or cathodal (negative), known as cathodal stimulation, which decreases neuronal excitability. In the context of PD, tDCS is utilized to improve motor function, reduce tremors, and enhance gait and balance, as well as to address cognitive deficits and depression associated with the condition. Studies have shown moderate improvements in motor and cognitive functions with tDCS, indicating its potential as a well-tolerated method with minimal side effects. However, it is important to note that the effects of tDCS are often temporary, requiring repeated sessions to maintain benefits. Additionally, not all patients respond equally to tDCS, underscoring the necessity for further research to determine the most effective stimulation parameters [32].

In a study investigating the effects of tDCS, Liu et al. conducted a systematic review to evaluate its efficacy as an adjunct therapy for patients with PD [33]. Their analysis revealed significant improvements in cognitive function, as indicated by scores on the Unified PD Rating Scale (UPDRS) I and the Montreal Cognitive Assessment (MoCA). However, the review found insufficient evidence to support the effectiveness of tDCS in enhancing motor function, based on UPDRS III scores and performance in tests such as the Timed Up and Go (TUG), Berg Balance Scale, and gait assessments. The authors emphasized the necessity for larger, multicenter trials to determine the optimal tDCS parameters for enhancing functional recovery in PD patients [33].

In summary, while tDCS shows potential for addressing cognitive functions and possibly improving motor functions in PD, its temporary effects and variability in patient responses underscore the need for further research to establish the most effective stimulation parameters and long-term benefits.

tACS involves applying an alternating electrical current to the scalp, allowing for frequency adjustments to target specific brain oscillations and modulate neural activity. In the context of PD, tACS is being explored for its potential to improve motor function by synchronizing brain oscillations, as well as enhancing cognitive function and mood. Like tDCS, tACS is generally well-tolerated with minimal side effects. However, research on tACS is still in the early stages and is so far less extensive compared to tDCS, necessitating further investigation to confirm its efficacy in PD. Additionally, patient responses to tACS can vary widely, emphasizing the need for more comprehensive studies to optimize its use [34]. Teo et al. (2017) conducted a study focusing on the effects of tACS on neural entrainment in both healthy individuals and those with PD [35]. They highlighted the early stage of research into the therapeutic potential of tACS and emphasized the gaps in understanding the causal relationship between neural oscillation dysfunctions and specific motor and cognitive deficits observed in PD [35]. While research by Krause et al. (2021) [36] indicated improvements in resting tremors and movement variability in PD, Teo et al. [35] pointed out that cardinal motor symptoms such as bradykinesia, rigidity, and gait disturbances likely involve distinct underlying mechanisms. Their findings underscored the necessity for further investigation into frequency-specific forms of NIBS to better elucidate their therapeutic benefits in PD [36]. Thus, tACS holds promise for improving motor function and cognitive outcomes in PD, but further research is essential to confirm its efficacy and understand its specific therapeutic potential.

tRNS applies a random electrical noise current to the scalp, which is believed to enhance cortical excitability and improve neural plasticity in PD patients. This stimulation may lead to improvements in motor function, cognition, and mood, potentially enhancing neuroplasticity and facilitating motor learning. Like other tES methods, tRNS is generally safe and well-tolerated; however, it is a relatively new technique with limited research in the context of PD. More studies are needed to determine the most effective parameters and protocols for its application [37].

Monastero et al. (2020) investigated the effects of tRNS applied over the primary motor cortex (M1) in patients with PD and mild cognitive impairment (PD-MCI) [38]. Their findings revealed significant improvements in motor ability, particularly in UPDRS-Motor Examination (UPDRS-ME) scores, following real tRNS compared to sham stimulation. While the study confirmed the safety and effectiveness of single-session tRNS over the left M1 for enhancing motor function in PD-MCI patients, it did not demonstrate significant improvements in executive functioning. The authors emphasized the necessity for further research utilizing multi-session tRNS targeting multiple brain areas, such as the dorsolateral prefrontal cortex and M1. They also highlighted the need for randomized controlled trials with larger sample sizes and standardized protocols to validate these findings and explore broader therapeutic implications [38]. Therefore, while tRNS shows promise in enhancing motor functioning in PD patients with mild cognitive impairment, further research employing multi-session tRNS with larger cohorts and standardized methods is essential to confirm and expand its therapeutic potential.

#### 3.3.3. Transcranial Focused Ultrasound Stimulation (tFUS)

Focused Ultrasound Stimulation or FUS is an emerging NIBS technique that has shown promise in treating various neurological disorders, including PD [39]. Its ability to precisely target specific brain regions offers potential advantages over other non-invasive stimulation methods [39]. tFUS utilizes focused ultrasound waves to modulate neuronal activity through mechanical effects, which involve mechanical vibrations that can stimulate or inhibit neurons, and thermal effects, which produce localized heating that can influence neuronal function [40]. tFUS targets specific motor circuits in the brain to alleviate symptoms such as tremors, rigidity, and bradykinesia, which are hallmark motor symptoms of PD [38]. Additionally, tFUS may have the potential to address NMSs associated with PD, including cognitive impairments and mood disorders. One of the key benefits of tFUS is its ability to target specific brain regions with millimeter precision, minimizing effects on surrounding tissues. Furthermore, tFUS is non-invasive, thereby avoiding the risks associated with surgical interventions. Some studies even suggest that tFUS can lead to long-lasting improvements in symptoms [40].

However, it is important to note that tFUS is still in the early stages of research for PD, and further clinical trials are necessary to establish its efficacy and safety. The application of tFUS requires advanced imaging techniques and precise targeting, which can pose technical challenges and may be costly. As with other brain stimulation techniques, patient responses to tFUS can vary [41]. Several studies have investigated the effects of focused ultrasound stimulation (FUS) on outcomes in PD. For example, Sinaia et al. (2022) reported on the long-term efficacy and safety of magnetic resonance imaging-guided focused ultrasound (MRgFUS) VIM-thalamotomy for treating tremors in tremor-dominant PD patients [42]. In a follow-up period ranging from 1 to 5 years involving 26 patients, significant reductions in tremor severity were observed, assessed by the Clinical Rating Scale for Tremor and the UPDRS. Most patients experienced substantial relief from tremors, with adverse events reported as mild and resolving within three months. Notably, the treatment also delayed the initiation of levodopa therapy in some patients, underscoring its long-term effectiveness and safety [42].

In a review by Krishna et al. (2017), advancements and applications of MRgFUS for various neurological conditions were discussed [43]. Initially approved for treating refractory essential tremors, FUS is being explored for its potential in managing PD, dystonia, neuropathic pain, obsessive-compulsive disorder (OCD), epilepsy, and brain tumors. Innovations in transducer design and electronic phase correction have facilitated precise brain lesioning and treatment monitoring through live anatomical thermography. Emerging applications for FUS include targeted drug delivery and neuromodulation. The non-invasive nature of FUS makes it a suitable option for patients who are ineligible for conventional surgical interventions, and future improvements are anticipated to enhance both its safety and efficacy [44].

Moreover, Schlesinger et al. (2017) reviewed the clinical use and treatment outcomes of MRgFUS for PD patients with medication-resistant symptoms [44]. Their findings indicated that MRgFUS demonstrated significant benefits in most patients, with only a few transient adverse events reported. However, the optimal target for lesioning appears to vary across treatment centers. While some centers target the pallidothalamic tract or thalamus to alleviate tremors and motor complications, the long-term efficacy and potential adverse events require further investigation. The study highlighted challenges associated with focusing ultrasound rays and targeting within the pallidum, suggesting that specialized centers experienced in the procedure may provide better outcomes [44].

In this context, tFUS shows promise in precisely targeting specific brain regions to address motor symptoms, cognitive impairments, and mood disorders associated with PD [5]. However, further studies are needed to establish its efficacy and safety and to refine patient selection and treatment targets. Additionally, the potential of detecting vagal nerve pathology as an early marker of PD and utilizing vagus nerve neuromodulation to treat early symptoms has been explored [5]. This approach could help rebalance autonomic dysfunction and improve clinical outcomes, emphasizing the importance of early detection and neuromodulation in managing neurodegenerative disorders [5].

#### 3.3.4. Transcutaneous Vagus Nerve Stimulation (tVNS)

tVNS is a non-invasive neuromodulation technique that involves stimulating the vagus nerve through the skin. tVNS delivers electrical impulses to the auricular branch of the vagus nerve, which transmits signals to the brainstem and various brain regions. This stimulation is believed to modulate neural circuits involved in motor control, cognition, and mood regulation. tVNS has been explored for its potential therapeutic effects in various neurological and psychiatric disorders, including PD. Specifically, tVNS aims to alleviate motor symptoms such as tremors, rigidity, and bradykinesia by influencing neural pathways involved in motor control. It is also being investigated for improving cognitive functions and alleviating mood disorders associated with PD NMSs [45].

tVNS is considered safe and non-invasive, with minimal side effects compared to invasive techniques. By targeting the vagus nerve, tVNS may modulate neural activity in brain regions affected by PD. Clinical studies suggest that tVNS may lead to improvements in motor symptoms and possibly NMSs [5]. However, responses to tVNS can vary among individuals, necessitating personalized treatment approaches. The optimal stimulation parameters (e.g., intensity, frequency, duration) are still being refined through ongoing research. Additionally, long-term effects and the maintenance of benefits require further investigation [46].

### 3.4. Invasive Technologies

Invasive brain stimulation technologies, such as DBS, responsive neurostimulation (RNS), intracortical micro-stimulation (ICMS), and cortical stimulation, offer targeted approaches to modulate neural circuits and alleviate symptoms in PD [47]. While these methods are effective in improving motor symptoms, they involve surgical risks and may have limited impact on NMSs. Ongoing research aims to optimize these techniques and explore their broader applications in neurological disorders [48]. IBS technologies involve procedures where devices are implanted directly into the brain or on its surface to modulate neural activity [49]. These techniques are often considered when non-invasive methods, such as transcranial stimulation, have not provided sufficient relief [50].

- DBS is the most established of these methods, involving the implantation of electrodes in specific brain regions to deliver continuous electrical stimulation. DBS has been shown to significantly alleviate motor symptoms in PD patients, such as tremors, rigidity, and bradykinesia, but its effects on NMSs remain less clear [51].
- RNS involves implanting a device that monitors brain activity and delivers electrical stimulation in response to abnormal patterns. Although primarily used for epilepsy, RNS is being explored for PD, offering real-time intervention that may reduce side effects compared to continuous stimulation [52].
- Intracortical Micro-stimulation (ICMS) involves the implantation of microelectrodes into the cerebral cortex to stimulate specific neurons, providing precise control over neural activity. However, ICMS is highly invasive and comes with significant risks [53].
- Cortical Stimulation entails placing electrodes on the brain’s surface to modulate cortical activity. This technique has been used experimentally to improve both motor and cognitive symptoms, but it also requires invasive surgery and has limited research specifically in PD [54].

#### Deep Brain Stimulation (DBS)

DBS is a well-established treatment for PD that offers significant relief from motor symptoms and improves the quality of life for many patients. While the procedure involves surgical risks and necessitates ongoing management, DBS remains a valuable therapeutic option, particularly for individuals with advanced PD or medication-refractory symptoms [55]. The process involves surgically implanting electrodes into specific brain regions, typically the subthalamic nucleus (STN), globus pallidus internus (GPi), or thalamus. DBS delivers electrical impulses to modulate abnormal neuronal activity, primarily alleviating motor symptoms such as tremors, rigidity, bradykinesia, and motor fluctuations in patients whose symptoms are not adequately controlled with medication. Clinical evidence demonstrates significant improvements in both motor symptoms and QOL, often allowing for reduced medication dosages, which leads to enhanced motor function, increased independence, and improved mobility [56].

The surgical procedure is typically conducted in two stages. The first stage involves the implantation of electrodes into the brain under local anesthesia and neuroimaging guidance, while the second stage entails the implantation of a pulse generator under the skin near the collarbone [57]. DBS provides consistent and adjustable symptom control, with lasting improvements over the years, frequently resulting in lower medication doses and reduced side effects [58]. Numerous studies have emphasized the benefits of DBS in treating PD. For instance, Malvea (2022) reviewed the condition, highlighting the motor and non-motor impairments resulting from dopamine loss in deep brain structures [59]. Although no cure exists, various pharmacological and surgical treatments, particularly DBS, have been developed to manage symptoms effectively. This review examined the DBS procedures, their challenges, and efforts to optimize DBS through brain mapping, smart DBS systems, and advanced electrode designs, underscoring the need for further research to enhance its effectiveness and patient-friendliness [59].

In a narrative review by Montemurro et al. (2022), the authors focused on new targets and technologies in DBS surgery for PD [21]. They emphasized that while traditional DBS targets like the subthalamic nucleus (STN) and globus pallidus internus (GPi) are effective for alleviating motor symptoms, they often fall short in addressing non-dopaminergic symptoms and may lead to associated side effects. The review highlighted emerging targets in DBS that show promise for improving a broader spectrum of PD symptoms. Although these areas require further evidence for widespread clinical application, they offer potential when combined with classical targets to enhance symptom control. Montemurro et al. also discussed advancements in device development, noting trends towards more personalized and minimally invasive options that could reduce costs and complications [21]. Beyond DBS, the review explored rapid advancements in biological therapies, gene therapy, cell engineering, tissue engineering, and biomaterials. These innovative approaches aim not only to improve symptom management but also to modify disease progression. However, the authors underscored the ethical considerations and risks associated with such therapies. They suggested that future directions in PD treatment might move towards regenerative neurosurgery, which aims not just to manage symptoms but also to potentially halt or reverse disease progression. Furthermore, they advocated for continued research into multimodal treatments that integrate technological advancements with a deep understanding of neuroanatomy for more effective therapeutic outcomes in PD [21].

Additionally, Krauss et al. (2021) discussed the evolution and future of DBS as a neurosurgical procedure used to modulate brain circuits in conditions like PD, essential tremor, and dystonia [36]. Modern DBS systems, adapted from cardiac technology, include an intracranial electrode, an extension wire, and a pulse generator. Advances in engineering, imaging, and our understanding of brain disorders are poised to enhance DBS efficacy and tolerability through innovations in electrode and battery design, stimulation paradigms, and sensing technologies. The review provides a comprehensive overview of DBS technology, highlighting the importance of ethical, privacy, and security considerations alongside these advancements. The authors predicted a future where DBS is safer, less invasive, more accurate, and effective, benefiting a broader range of patients with neurological and psychiatric disorders [36]. This overview underscores DBS as a cornerstone therapy for managing PD, emphasizing its mechanisms, applications, efficacy, and considerations for patients considering this treatment option. However, challenges remain. Surgical risks include infection, bleeding, and hardware-related complications. Moreover, programming and optimizing stimulation parameters require specialized expertise, and NMSs such as cognitive deficits and mood disorders may not experience significant improvement [60].

### 3.5. Clinical Utilization of Brain Stimulation

Brain stimulation techniques are increasingly employed in the management of PD symptoms that are not adequately controlled by medication alone. These techniques aim to modulate neural activity in specific brain regions to alleviate both motor and, in some cases, NMSs associated with PD. They present promising alternatives for symptom management, particularly when pharmacological treatments fall short [61]. Ongoing research aims to optimize these brain stimulation methods, expand their clinical applications, and enhance our understanding of their long-term effects on symptom management and quality of life in individuals with PD [62]. In this context, we categorize the applications of various brain stimulation techniques in treating both motor and NMSs of PD.

#### 3.5.1. Clinical Application of Brain Stimulation in Motor Symptoms

PD is characterized by a variety of motor symptoms, including tremors, gait disturbances, bradykinesia, postural instability, and rigidity [5]. Among these, tremor is a prominent symptom, presenting as involuntary rhythmic shaking of a limb or other body parts, while rigidity is characterized by stiffness and resistance to limb movement. Brain stimulation techniques, such as DBS, TMS, and tFUS, have emerged as promising strategies for alleviating these symptoms, enhancing mobility, and improving overall quality of life for individuals with PD [63]. These techniques provide targeted approaches to address gait disturbances, bradykinesia, and postural instability in PD patients, particularly when medications alone are insufficient. By modulating neural circuits involved in motor control, brain stimulation methods contribute to significant improvements in mobility, coordination, and QOL [64]. Ongoing research aims to refine these techniques, optimize treatment protocols, and explore their broader applications in enhancing motor function and QOL for PD patients experiencing tremors.

Brain stimulation techniques play a crucial role in managing motor symptoms for PD patients who do not achieve adequate symptom control with medication. They target neural circuits involved in motor function, offering targeted therapeutic benefits. Research continues to expand their clinical applications, optimize treatment protocols, and assess their long-term efficacy and safety in the management of PD [64]. The effectiveness of these brain stimulation techniques in reducing tremors has been documented across various neurological disorders, as evidenced by numerous studies.

França (2018) emphasized the role of the cerebellum in movement disorders and its increasing significance in neuromodulation [65]. Their results demonstrated improvements in motor symptoms with generally safe outcomes in movement disorders like PD, Amyotrophic Lateral Sclerosis, Huntington’s disease, Dystonia, Tic disorders, and Essential Tremor, though minor side effects like headache, skin erythema, and rare infections were noted. This review underscores the potential of cerebellar modulation in enhancing symptom management across these disorders, prompting the need for further research to advance this promising therapeutic approach [65].

In another narrative review, Pateraki et al. (2022) describe TMS as a non-invasive method that utilizes a magnetic field generated by passing electric current through a coil placed on the scalp [64]. TMS can be applied singly, in pairs, or repetitively (rTMS), affecting brain activity by inducing long-term potentiation or depression [61]. Low frequencies (≤1 Hz) typically suppress cortical excitability, while higher frequencies (>1 Hz) enhance it. This technique is generally safe with mild side effects and has been explored as a potential treatment for various neurodegenerative diseases, including movement disorders. Despite limited approval in current guidelines, rTMS shows promise in treating aspects of movement disorders such as PD, Amyotrophic Lateral Sclerosis, Huntington’s disease, Dystonia, Tic disorders, and Essential Tremor. The review synthesizes existing literature on rTMS applications in these disorders, highlighting its potential therapeutic benefits and calling for further research to expand its clinical use and optimize treatment protocols. However, larger, well-designed clinical trials are necessary to establish the sustained efficacy of rTMS, optimize stimulation protocols, and determine the ideal targets, intensity, duration, and frequency of sessions for treating gait impairments in PD [64].

In addition, Nardone et al. (2020) discussed TMS as a valuable tool for studying motor impairments in PD and exploring therapeutic interventions [25]. HF-rTMS, particularly when applied bilaterally over motor cortical areas, shows promise in improving motor symptoms in PD. However, research on rTMS effects specifically on FOG and other gait disturbances in PD remains limited. The review highlights that combining rTMS with treadmill training enhances therapeutic outcomes, and using an H-coil allows stimulation of deeper brain regions like the medial prefrontal cortex, potentially aiding future therapies. In contrast, theta burst stimulation has not proven effective for treating gait disturbances in PD. The review proposes dual-mode NIBS, such as preconditioning motor cortex rTMS with tDCS, as a novel approach for PD patients with gait issues. Recent studies suggest targeting the supplementary motor area for brain stimulation in FOG treatment [25,66].

Handwriting difficulties are common in PD due to motor symptoms such as bradykinesia, rigidity, and tremor [67]. Bradnam et al. (2015) explore the role of the cerebellum in primary focal hand dystonia (FHD) and its potential as a target for NIBS [68]. The study aimed to assess whether cerebellar tDCS could enhance handwriting and cyclic drawing kinematics in individuals with hand dystonia by modulating cerebellar-brain inhibition (CBI) evoked by TMS. In the study, eight participants with dystonia (five with writer’s dystonia and three with musician’s dystonia) and eight age-matched controls underwent anodal, cathodal, and sham cerebellar tDCS in separate sessions. Dystonia severity was evaluated using the Writer’s Cramp Rating Scale (WRCS) and the Arm Dystonia Disability Scale (ADDS). Kinematic measures, such as stroke frequency during handwriting and cyclic drawing, and pen pressure during drawing tasks, were used to assess motor function. The findings indicated that anodal cerebellar tDCS led to improvements in handwriting stroke frequency and pen pressure, as well as increased speed during fast cyclic drawing tasks. However, the study did not establish a clear neurophysiological mechanism underlying these improvements. The study concludes that cerebellar anodal tDCS shows promise in enhancing motor tasks affected by hand dystonia. Further research involving larger, more homogeneous populations is recommended to better understand the therapeutic potential and refine the application of cerebellar tDCS in dystonia treatment [68].

Fatigue is also a common and impactful symptom in PD, often present in the early stages but potentially occurring at any point, irrespective of the severity of movement symptoms. Zaehle et al. (2021) highlighted the substantial impact of PD-related fatigue (PDRF) on quality of life and the limited treatment options available [69]. They have proposed frontal anodal tDCS as a promising therapeutic approach due to its ability to modulate cortical excitability, particularly in the frontal cortex, which is implicated in fatigue mechanisms. While existing research supports the efficacy of frontal anodal tDCS in reducing fatigue in various neurological conditions, further studies are necessary to optimize stimulation parameters, validate treatment outcomes objectively, and explore the feasibility of home-based applications [69]. Therefore, brain stimulation techniques have shown promise in alleviating motor symptoms and enhancing the QOL for individuals with PD, and ongoing research continues to refine these techniques and explore their broader applications in improving motor function and QOL to effectively establish their clinical utility.

#### 3.5.2. Clinical Application of Brain Stimulation in Non-Motor Symptoms

NMSs in PD encompass a wide range of manifestations beyond motor dysfunction, including cognitive impairment, mood disorders, autonomic dysfunctions, sleep disturbances, sensory symptoms, and gastrointestinal issues. Brain stimulation techniques, including DBS, TMC, tDCS, and tFUS, have been investigated for their potential to alleviate these symptoms and improve overall quality of life in PD patients. These techniques target neural circuits involved in NMSs, aiming to alleviate symptoms and enhance the overall QOL for individuals with PD [70].

##### Cognitive Impairments

Cognitive impairment is a significant non-motor symptom in PD, affecting aspects such as memory, executive function, attention, decision-making, recall, perception of time, visuospatial abilities, and language. Additionally, dementia can be a severe cognitive complication in PD, characterized by a progressive decline in memory, thinking, and reasoning abilities [45]. Brain stimulation techniques have been investigated for their potential to alleviate cognitive decline and improve the quality of life in PD patients with dementia. These techniques target neural circuits involved in cognitive function, mood regulation, and autonomic control, aiming to alleviate symptoms and enhance overall QOL for PD patients [69,70,71].

In a study by Ouellet et al. (2018), the effects of tDCS applied bilaterally over the orbitofrontal cortex (OFC) on decision-making and cognitive impulse control in healthy subjects were investigated [71]. The OFC plays a crucial role in these processes within a neural network involving multiple cortical and subcortical regions. The study included 45 healthy participants who were randomly assigned to receive either active or sham anodal tDCS sessions (1.5 mA) over either the left or right OFC, coupled with contralateral cathodal tDCS. Participants underwent a series of computerized tasks both before and after the tDCS session to assess decision-making, cognitive impulse control, mood, attention, and motor impulse control. The findings revealed that participants who received active anodal tDCS, regardless of the stimulation site, demonstrated improvements in decision-making and enhanced cognitive impulse control. However, tDCS did not significantly affect mood, attentional levels, or motor impulse control based on the tasks administered. These results suggest that bilateral anodal tDCS over the OFC can modulate higher cognitive functions related to decision-making and impulse control in healthy individuals, offering potential for therapeutic interventions targeting conditions characterized by impaired decision-making and impulse control, such as addiction and suicidal behavior [71].

Furthermore, Sanches et al. (2021) reviewed the potential of NIBS techniques like TMS and tDCS in managing cognitive decline from neurodegenerative diseases [72]. They highlighted global concerns over the rising burden of neurodegenerative diseases and emphasized the need for robust clinical trials to validate the efficacy of NIBS. Despite promising initial findings, inconsistencies in study protocols and biomarker utilization hinder conclusive therapeutic outcomes. The review called for standardized protocols, improved biomarkers, and personalized treatment approaches to maximize the benefits of NIBS. Future research should focus on refining stimulation techniques and understanding their neurophysiological impacts across different neurocognitive conditions [72].

Suarez-García et al. (2020) conducted a systematic review and meta-analysis on the efficacy of tDCS for treating cognitive deficits in PD, highlighting the strong effects of anodal tDCS on executive functions post-stimulation, while calling for improved methodologies and further research to optimize treatment protocols [73]. Elder et al. (2019) conducted a trial to evaluate the effects of consecutive sessions of tDCS on visual hallucinations in Lewy body dementia (LBD), concluding that this tDCS protocol did not effectively mitigate visual hallucinations or alter related cognitive functions in LBD [74].

##### Neuropsychiatric Symptoms

Neuropsychiatric symptoms in PD encompass a range of behavioral and psychological changes, including depression, anxiety, apathy, and impulse control disorders [75]. Depression is a common and challenging neuropsychiatric symptom in PD, significantly impacting patients’ quality of life. Psychosis is another significant neuropsychiatric complication in PD, characterized by hallucinations and delusions. Brain stimulation techniques have been explored for their potential to alleviate psychotic symptoms in PD patients. Hallucinations, often affecting visual perception, are a common and distressing symptom in PD. 

##### Impulse-Control Disorders

Impulse-control disorders (ICDs) in PD are characterized by problematic behaviors resulting from impulsivity, often leading to actions that are harmful or detrimental. These disorders can manifest as compulsive gambling, shopping, eating, or hypersexuality. Ouellet et al. (2018) conducted a study exploring the impact of tDCS on decision-making and impulse control processes mediated by the orbitofrontal cortex (OFC) [71]. The findings indicated that active tDCS enhanced decision-making abilities, as evidenced by increased scores on the Iowa Gambling Task, and improved cognitive impulse control, demonstrated by reduced interference in the Stroop Word-Color Task. These results suggest the potential for tDCS as a non-invasive therapeutic approach for psychiatric conditions linked to impaired decision-making and impulse control [76,77].

##### Other Symptoms

Sleep disorders are common in PD, affecting various aspects such as Rapid Eye Movement (REM) behavior disorder and insomnia. Brain stimulation techniques have been investigated for their potential to manage these disturbances. In a systematic review, Babiloni et al. (2021) highlighted the significance of sleep disturbances across neurological and neuropsychiatric conditions, impacting well-being and quality of life [78]. While current treatments, including medications and cognitive behavioral therapy, often present limitations in effectiveness and side effects, the review suggested that rTMS and tDCS are generally safe and show promise in improving insomnia symptoms and sleep quality in these populations. However, due to the inclusion of studies with a high risk of bias, these findings should be interpreted cautiously. Future research is recommended to reduce bias, improve study quality, and optimize stimulation parameters, aiming to enhance the efficacy of brain stimulation techniques in managing sleep-related issues alongside conventional treatments [77].

Urinary incontinence and altered sexual function are notable NMSs in PD, impacting patients’ quality of life. Smith et al. (2021) conducted a systematic review on neuromodulation techniques for treating bladder symptoms in PD, analyzing ten primary studies that focused on transcutaneous or percutaneous tibial nerve stimulation (TNS), sacral neuromodulation (SNM), and TMS [79]. The studies generally reported positive outcomes, particularly with TNS showing benefits across a range of measures. However, concerns regarding placebo effects and the limited number of well-controlled studies were noted. Only two randomized sham-controlled trials for TNS demonstrated superiority over sham treatment, highlighting a need for further rigorous research. The authors emphasize the importance of robust clinical trials to definitively establish the efficacy of neuromodulation in managing PD-related bladder symptoms, potentially offering a medication-free alternative with fewer side effects [79].

### 3.6. Safety of Brain Stimulation

Ensuring the safety of brain stimulation techniques is paramount, particularly in conditions like PD. While these techniques offer promising therapeutic benefits, careful patient selection, precise targeting, treatment monitoring, and adherence to ethical and regulatory standards are essential to mitigate risks and optimize outcomes. Advances in technology continue to improve the safety and efficacy of these techniques, broadening their applications in neurological and psychiatric disorders.

NIBS techniques such as TMS, tES, and tFUS generally have favorable safety profiles compared to invasive approaches like DBS. When administered correctly, TMS is safe and well-tolerated, with mild discomfort or headaches being common side effects and rare seizure risks, particularly in individuals with a history of seizures. tDCS has minimal risks, typically limited to tingling or mild headaches, though improper electrode placement may lead to skin burns, emphasizing the need for precise application techniques. tFUS provides targeted brain stimulation with minimal risk to surrounding tissues, though potential side effects like transient headaches or mild cognitive changes require close monitoring for safety. Regulatory bodies oversee these methods to ensure safety standards are met, while ethical guidelines safeguard patient consent and privacy in both research and clinical settings [80,81].

In contrast, IBS techniques, such as DBS, involve the surgical implantation of electrodes directly into specific brain regions. DBS offers significant therapeutic benefits for conditions like PD, but its invasive nature necessitates rigorous safety protocols due to potential risks, including infection, bleeding, adverse anesthesia reactions, and, in rare cases, stroke or neurological deficits. Accurate electrode placement is crucial to effectively target the intended brain structures while minimizing unintended effects. Adherence to patient selection criteria, precise surgical techniques, and thorough post-operative management is essential to optimize both safety and efficacy. Advances in surgical techniques, device technology, and monitoring continue to improve outcomes and expand the applications of IBS in treating various neurological and psychiatric disorders [82].

## 4. Discussion

Despite the encouraging findings associated with various brain stimulation techniques, further research is essential to optimize stimulation protocols, elucidate underlying mechanisms, and establish standardized clinical guidelines. The work of Sanches et al. (2021) and Suarez-García et al. (2020) emphasizes the necessity for rigorous clinical trials to substantiate the efficacy of non-invasive techniques in addressing cognitive decline and other NMSs prevalent in PD [72,73]. Non-invasive techniques, including TMS, tDCS, and tFUS, provide safer initial options with fewer risks; however, they may be limited in reaching deep brain areas and may not have the sustained efficacy offered by more invasive methods like DBS. DBS, despite its surgical nature, offers precise and effective symptom relief for severe motor impairments and deep brain disorders like PD, making careful consideration of surgical risks and long-term management essential. Ongoing technological advancements and research continue to improve safety profiles and broaden the therapeutic scope of these techniques in neurology and psychiatry. Advances in neuroimaging and computational modeling are enhancing our understanding of how brain stimulation affects neural circuits, guiding the development of more targeted interventions. Additionally, the integration of brain stimulation with other therapies, such as cognitive training and pharmacotherapy, holds promise for synergistic effects in treating complex disorders.

However, the efficacy of non-invasive techniques may be constrained by limitations in depth of stimulation and durability of effects. In contrast, DBS remains a benchmark therapeutic approach for patients with severe motor symptoms and treatment-refractory cases of PD. The precision with which DBS can target deep brain structures, such as the subthalamic nucleus (STN) or globus pallidus internus (GPi), allows for significant and sustained symptom alleviation, particularly in individuals unresponsive to conventional pharmacotherapy.

The selection between non-invasive and invasive approaches necessitates a comprehensive assessment of patient-specific factors, including symptom severity, overall health status, and historical treatment responses. Typically, non-invasive techniques are employed as initial interventions or for less severe manifestations of PD, whereas DBS is reserved for advanced stages of the disease where other treatment modalities have proven ineffective. This treatment paradigm underscores the importance of personalized therapeutic strategies tailored to the individual characteristics and needs of each patient.

Key aspects of non-invasive (TMS, tDCS, tFUS) and invasive (DBS) brain stimulation techniques are summarized in Table 1, Table 2, Table 3, Table 4, Table 5, Table 6 and Table 7.

These tables provide a concise overview of each technique’s key characteristics, helping clinicians and researchers understand their differences and potential applications in clinical practice. Non-invasive techniques generally offer a safer profile with lower risks of serious adverse events compared to DBS, which involves invasive surgery. DBS is highly effective for targeting deep brain structures precisely, making it particularly suitable for severe cases that are often unresponsive to other treatments. Non-invasive approaches are typically used as initial interventions or for managing milder symptoms, while DBS is reserved for cases where other treatments have not been effective.

## 5. Conclusions

Brain stimulation methods are powerful tools that have significantly advanced our understanding of the brain and offered new hope for patients with challenging neurological and psychiatric conditions. Continued research and technological innovations are expected to further expand their applications and improve their therapeutic outcomes. PD is typically classified into five stages, each with increasing severity of symptoms. The choice of NIBS methods such as TMS and TDCS can be tailored according to these stages. In Stage 1, tDCS is proposed due to its low risk and cost-effectiveness, helping modulate cortical excitability in early mild symptoms. For Stage 2, either tDCS or rTMS can be used, with tDCS continuing to enhance cortical plasticity and rTMS providing more targeted relief. By Stage 3, rTMS is more appropriate for significant symptom management due to its targeted stimulation. In Stage 4, rTMS is preferred for addressing severe motor symptoms with more frequent sessions needed. For Stage 5, rTMS remains the method of choice, often combined with other therapies for managing advanced disease symptoms. Throughout all stages, consultation with a neurologist or movement disorder specialist is crucial for tailoring treatment to individual needs.

When considering the costs associated with treatment options for PD patients, they vary significantly based on the type of intervention. Non-invasive techniques such as TMS are generally more affordable with lower equipment costs. However, TMS is still largely experimental for PD and primarily used to address depression associated with the disease. TDCS falls into a moderate cost category, involving higher expenses due to equipment and professional fees. It is currently employed in research settings to potentially enhance motor and cognitive functions, though it has not yet become a standard treatment for PD. DBS, on the other hand, represents a high-cost option due to surgical procedures and ongoing management. DBS is widely accepted and utilized for managing severe motor symptoms in advanced stages of PD, reflecting its precision and effectiveness in targeting deep brain structures.

Future research should focus on several key areas: (1) conducting large-scale, randomized controlled trials to validate the efficacy of NIBS techniques across diverse PD populations; (2) exploring the neurophysiological mechanisms underpinning both motor and non-motor symptom improvement; (3) refining stimulation parameters and protocols to enhance treatment outcomes; and (4) investigating the long-term effects and safety of these techniques in various patient cohorts. Additionally, interdisciplinary approaches integrating neurology, psychiatry, and rehabilitation sciences will be essential to develop comprehensive treatment strategies that address the multifaceted nature of PD.

## Figures and Tables

**Table 1:** Comparing different types of TMS (i.e., single pulse TMS, rTMS, and iTBS) [25,26,27,31,32].

Aspect	TMS	rTMS	iTBS
**Mechanism of Action**	Magnetic fields induce electrical currents in the brain to modulate neuronal activity.	Repeated magnetic pulses at specific frequencies lead to longer-lasting changes in brain activity.	Short bursts of HF stimulation patterned to mimic natural theta rhythms.
**Frequency**	Single pulses or varied frequencies.	HF (≥5 Hz) excites, LF (≤1 Hz) inhibits neural activity.	Typically around 50 Hz bursts with theta pattern (e.g., 5 Hz).
**Session Duration**	Varies (usually 20–40 min).	20–40 min per session.	Usually only a few minutes per session.
**Applications in PD**	Motor and NMSs (depression, cognitive impairments).	Motor symptoms (bradykinesia, tremors, rigidity) and NMSs (depression, anxiety).	Motor symptoms and potentially cognitive/mood improvements.
**Efficacy**	Moderate improvement in motor function and symptom relief.	Significant benefits in reducing motor symptoms and medication needs.	Similar or superior outcomes to rTMS in some studies, particularly in motor function.
**Duration of Effects**	Temporary, requires repeated sessions.	Longer-lasting than single TMS but still requires frequent sessions.	Potentially longer-lasting effects than rTMS.
**Patient Response Variability**	Variable response among patients.	Variable response among patients.	Variable response among patients.
**Cost and Accessibility**	Moderate, depends on availability.	High, depends on frequency of sessions and availability.	Potentially lower due to shorter session duration.
**Advantages**	Non-invasive, well-tolerated.	Effective for both motor and NMSs, reduces medication dosage.	Time-efficient, similar or superior efficacy, reduced treatment burden.
**Limitations**	Temporary effects, variable patient response.	Requires frequent sessions, high cost, variable response.	Long-term efficacy needs more research, variable response.

**Table 2:** Comparing different types of tES (i.e., tDCS, tACS, and tRNS) [52,68,69,76].

Aspect	tDCS	tACS	tRNS
**Mechanism of Action**	Constant, low-intensity current; anodal (+) increases excitability, cathodal (−) decreases excitability.	Alternating current; frequency-adjustable to target specific brain oscillations.	Random electrical noise current enhances cortical excitability and plasticity.
**Applications in PD**	Motor symptoms (tremors, gait, balance), cognitive and mood symptoms.	Motor function improvement by synchronizing brain oscillations, cognitive and mood enhancement.	Motor function, cognition, and mood improvement.
**Efficacy**	Moderate improvements in motor and cognitive functions.	Flexible frequency allows targeted modulation; potential cognitive and mood benefits.	Enhanced neuroplasticity and motor learning potential.
**Duration of Effects**	Temporary, requiring repeated sessions.	Benefits often short-lived, requiring repeated sessions.	Effects may be temporary, requiring more sessions.
**Patient Response Variability**	Variable response among patients.	Variable response among patients.	Variable response among patients.
**Cost and Accessibility**	Low cost, portable, and widely accessible.	Low cost, portable, and widely accessible.	Low cost, portable, and widely accessible.
**Advantages**	Non-invasive, safe, well-tolerated.	Safe, well-tolerated, adjustable frequency for targeted effects.	Safe, well-tolerated, potential for enhanced plasticity.
**Limitations**	Temporary effects, requires optimization of parameters.	Early research stage, requires more studies for PD efficacy.	Limited research, needs optimization of parameters.

**Table 3:** Transcranial Focused Ultrasound Stimulation (tFUS) [4,39,40,41,42,43,44,63,70].

Aspect	Transcranial Focused Ultrasound Stimulation (tFUS)
**Mechanism of Action**	Focused ultrasound waves create mechanical and thermal effects to modulate neuronal activity.
**Applications in PD**	Motor symptoms (tremors, rigidity, bradykinesia), potential for cognitive and mood improvements.
**Efficacy**	High-precision targeting, potential for long-lasting symptom improvement.
**Duration of Effects**	Potentially long-lasting effects, but more research is needed.
**Patient Response Variability**	Variable response among patients.
**Cost and Accessibility**	High cost and technical complexity, requiring advanced imaging and precise targeting.
**Advantages**	Non-invasive, high precision, potential for long-lasting effects.
**Limitations**	Early research stage, technical complexity, variable patient response.

**Table 4:** transcutaneous Vagus Nerve Stimulation (tVNS) [5,45,46].

Aspect	Transcutaneous Vagus Nerve Stimulation (tVNS)
**Mechanism of Action**	Stimulation of the vagus nerve through the skin, influencing brainstem and cortical regions.
**Applications in PD**	Motor symptoms (tremors, rigidity, bradykinesia), cognitive function, mood disorders.
**Efficacy**	Non-invasive, modulates neural pathways, potential for symptom improvement.
**Duration of Effects**	Short-term effects observed; long-term effects need further research.
**Patient Response Variability**	Responses vary among individuals.
**Cost and Accessibility**	Generally considered safe and accessible; costs may vary.
**Advantages**	Non-invasive, minimal side effects, potential for improving motor and NMSs.
**Limitations**	Variable response, optimal parameters need refinement, long-term efficacy requires more study.

**Table 5:** Comparing different types of non-invasive brain stimulations [5,25,39,40,41,42,43,44,51,56,57,58,59,60,61,62,63,64,66,72].

Aspect	TMS	tDCS	tFUS	DBS
**Mechanism**	Magnetic pulses induce electrical currents.	Low-intensity direct current alters neuronal excitability.	Ultrasound waves stimulate brain tissue.	Implanted electrodes deliver electrical impulses.
**Applications**	Depression, neuropathic pain, cognitive disorders.	Depression, chronic pain, cognitive disorders.	Essential tremor, neuropathic pain, brain tumors.	Parkinson’s, essential tremor, dystonia.
**Safety**	Generally safe; minor side effects like headache; rare risk of seizures.	Low risk; mild side effects like skin irritation, headache; risk of burns with improper use.	Precise targeting reduces risks; potential side effects include headache or mild cognitive changes.	Invasive surgery risks including infection, bleeding, hardware issues.
**Effectiveness**	Effective for milder symptoms and cognitive disorders.	Effective for enhancing learning, managing chronic pain.	Effective for essential tremor, neuropathic pain; limited data for other conditions.	Highly effective for severe motor symptoms, DBS.
**Suitability**	Outpatient setting; suitable for research applications.	Outpatient setting; suitable for research applications.	Outpatient setting; suitable for precise targeting.	Requires neurosurgical expertise; long-term management.
**Long-term Management**	Minimal maintenance; ongoing research in optimization.	Minimal maintenance; ongoing research in optimization.	Minimal maintenance; ongoing research in optimization.	Requires regular monitoring; battery replacements.
**Cost**	Moderate; varies by region and treatment protocols.	Low; equipment costs and session fees.	High initial cost; potential cost savings over time.	High initial and ongoing costs; varies by healthcare system.

**Table 6:** Comparing benefits and limitations of non-invasive and invasive brain stimulation [25,26,37,48,49,50,51,57,68,69,76].

Aspect	Non-Invasive Techniques (TMS, tES, tFUS)	Invasive Technique (DBS)
**Targeting Depth**	Limited depth, mainly cortical; difficult to reach deep brain structures.	Direct deep brain targeting (e.g., STN, GPi in PD).
**Procedure**	Non-surgical; applied externally.	Surgical implantation of electrodes in specific brain regions.
**Side Effects**	Mild (e.g., headaches, tingling, transient cognitive changes).	Higher risks (e.g., infection, bleeding, anesthesia reactions).
**Recovery Time**	Minimal; patients can typically resume normal activities shortly.	Longer recovery due to surgical nature, with possible rehabilitation.
**Efficacy for Severe Symptoms**	Effective for mild-to-moderate symptoms; may have limited effect on severe cases.	Highly effective for severe symptoms, especially motor symptoms in PD.
**Long-Term Efficacy**	May require repeated sessions for sustained benefit.	Long-lasting, with adjustable settings for symptom management.
**Monitoring Requirements**	Periodic monitoring during sessions, minimal ongoing monitoring.	Continuous monitoring and device management needed post-surgery.
**Patient Suitability**	Suitable for a broad range, especially patients avoiding surgery.	Suitable for those with severe symptoms not managed by other means.
**Regulatory Oversight**	Generally classified as low risk; guided by regulatory standards.	Highly regulated with stringent safety and ethical protocols.
**Research & Development**	Ongoing, focusing on optimization of stimulation parameters.	Advances in device technology and surgical precision.

**Table 7:** Comparing advantages, disadvantages, and applications of non-invasive and invasive brain stimulation.

Method	Cost	Advantage	Disadvantage	Application for PD Patients
TMS	Moderate	Non-invasive, relatively safe, and has minimal side effects.	Short-lived effects requiring repeated sessions.	Experimental for motor symptoms and depression in PD.
tES	Low	Non-invasive, portable, and easy to administer.	Mechanisms not fully understood, optimal parameters unclear.	Potential to enhance motor and cognitive function in PD.
tVNS	Moderate	Non-invasive, may improve autonomic and motor function.	Limited clinical evidence, potential discomfort.	Experimental for motor symptoms and mood regulation in PD.
tFUS	Moderate	Non-invasive, focused delivery, precise targeting.	Expensive equipment, limited availability.	Emerging use for targeted brain modulation in PD.
DBS	High	Significant and sustained therapeutic benefits.	Invasive with potential risks like infection and hardware complications.	Established treatment for motor symptoms in PD.

## Nomenclature

**Abbreviation**	**Full Form**	**Abbreviation**	**Full Form**
DBS	Deep Brain Stimulation	rTMS	Repetitive Transcranial Magnetic Stimulation
FDA	Food and Drug Administration	SNM	Sacral Neuromodulation
IBS	Invasive Brain Stimulation	tACS	Transcranial Alternating Current Stimulation
ICDs	Impulse-Control Disorders	tDCS	Transcranial Direct Current Stimulation
ICMS	Intracortical Macrostimulation	tES	Transcranial Electrical Stimulation
iTBS	Intermittent Theta Burst Stimulation	tFUS	Transcranial Focused Ultrasound Stimulation
MRgFUS	MRI-guided Focused Ultrasound Stimulation	TMS	Transcranial Magnetic Stimulation
NIBS	Non-Invasive Brain Stimulation	TNS	Tibial Nerve Stimulation
PD	Parkinson’s Disease	tRNS	Transcranial Random Noise Stimulation
PD-MCI	Parkinson’s Disease with Mild Cognitive Impairment	tVNS	Transcutaneous Vagus Nerve Stimulation
QOL	Quality of Life	UPDRS	Unified Parkinson’s Disease Rating Scale
